# Effect of antiretroviral therapy on changes in the fertility intentions of human immunodeficiency virus-positive women in Addis Ababa, Ethiopia: a prospective follow-up study

**DOI:** 10.4178/epih.e2017028

**Published:** 2017-07-16

**Authors:** Hussen Mekonnen, Fikre Enquselassie

**Affiliations:** 1 Department of Nursing and Midwifery, School of Allied Health Sciences, College of Health Sciences, Addis Ababa University, Addis Ababa, Ethiopia; 2 Department of Preventive Medicine, School of Public Health, Addis Ababa University, Addis Ababa, Ethiopia

**Keywords:** Fertility, Follow-up studies, Human immunodeficiency virus, Women, Antiretroviral therapy, Ethiopia

## Abstract

**OBJECTIVES:**

With access to antiretroviral therapy (ART), people living with human immunodeficiency virus (HIV) are able to consider childbearing to a greater extent than previously. In many cases, ART has transformed their intentions to have children. The present study aimed to assess changes in fertility intentions 12 months after ART initiation among HIV-positive women in Addis Ababa, Ethiopia.

**METHODS:**

An institution-based follow-up study was conducted among 360 HIV-positive women in Addis Ababa. A logistic regression model was used to assess the influence of socio-demographic, reproductive health, and clinical characteristics on changes in the fertility intentions of women.

**RESULTS:**

Overall, 40.8% (147 of 360) of the women reported that they desired to have a child in the future at the baseline visit, while 48.3% (174 of 360) did so at the 12-month follow-up. The proportion of women who reported that they desired to have a child 12 months after ART initiation was higher among ART-initiated women (55.8%, 106 of 190) than ART-naïve women (40.0%, 68 of 170). The adjusted analysis indicated that a change in fertility intentions between baseline and the follow-up visit was significantly associated with ART use (adjusted odds ratio [aOR], 2.47; 95% confidence interval [CI], 1.20 to 5.20) and marital status, with single (aOR, 5.33; 95% CI, 1.10 to 25.92) and married (aOR, 6.35; 95% CI, 1.44 to 27.99) women being more likely to report fertility intentions than divorced/widowed women.

**CONCLUSIONS:**

ART use was a significant predictor of change in fertility intentions between the baseline and follow-up visit, which suggests that additional efforts are necessary to integrate family planning and HIV services to address the safe fertility goals of women in the study area.

## INTRODUCTION

Sub-Saharan Africa is the region most severely affected by human immunodeficiency virus (HIV) [1]. Women of reproductive age account for 58.0% of people living with HIV [2] and 53.0% of all adult deaths [3]. In Ethiopia, more women (2.9%) than men (1.9%) are living with HIV [4]. Most of these women are particularly vulnerable to HIV due to the complex burdens they face, including physiological and social vulnerability and gender inequalities [3,5]. Since these infected women are of childbearing age [4], they risk infecting their children, and thus face difficult choices about childbearing.

Despite the risks and challenges, studies from sub-Saharan Africa have revealed that many HIV-positive women continue to desire children even with knowledge of their HIV-positive status [6-8]. Studies have reported that HIV does not seem to negatively modify subsequent childbearing intentions [9,10], and some have reported that the majority of respondents intended to have more than 2 children [11,12]. The desire to have children in the future has significant implications for the transmission of HIV to an individual’s sexual partner and to the newborn [13,14]. However, with access to antiretroviral therapy (ART) and preventive care, people living with HIV are able to consider childbearing to a greater extent than was previously possible [15]. Previous studies involving women on ART have revealed that most pregnancies among HIV-positive women may be intentional [16-18]. Subsequent studies from sub-Saharan Africa have reported that ART access influenced the fertility intentions of HIV-positive women [10,19,20].

Studies from different parts of Africa have reported that the pregnancy rate of women on ART was significantly higher than that of ART-naïve women [21]. A study from South Africa stated that ART increased the fertility desire of couples over time [6] and that the pregnancy rate among women who recently initiated ART with a low CD4 count and a high viral load was high [22]. Furthermore, women on ART quickly became pregnant even before their CD4 count improved, both because they wanted to fulfill their social role as women and because they wanted to prove that they were healthy [23].

All these findings indicate that ART has not only transformed HIV-positive women’s physical state, but also transformed mostly what had been desire into intention [24]. Since childbearing intentions are a predictor of women’s subsequent fertility behaviors [25], developing reproductive health services that are responsive to HIV-positive women in relation to ART requires a clear understanding of their expressed childbearing intentions. However, it may be difficult to sustain fertility intentions over time, as they are affected by women’s changing life situations, and it is therefore difficult to capture such issues by cross-sectional studies conducted within an area. Furthermore, efforts are under way in Ethiopia to prevent mother-to-child transmission of HIV [26,27]. It is worthwhile to update such efforts and to provide information about the present situation regarding fertility intentions, as well as change in fertility intentions over time. Thus, the purpose of this study was to assess changes in the fertility intention of HIV-positive women in Addis Ababa 12 months after ART initiation.

## MATERIALS AND METHODS

### Study area

The study was conducted in public health facilities of Addis Ababa, Ethiopia. In Ethiopia, fee-based ART began in 2003 and free ART was launched in 2005 [28]. Since then, 5 public hospitals and 25 health centers have been offering ART [29]. Approximately 124,983 people living with HIV have been enrolled in Addis Ababa, of whom 76,035 have started ART and 54,667 are currently on ART [4]. The fertility rate is 2.4 children per reproductive-age woman in Addis Ababa [4].

### Study design and sampling procedures

**Figure 1.**
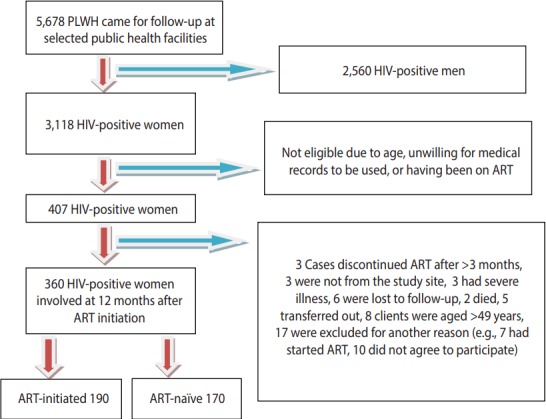
Schematic representation of the enrollment of subjects in Addis Ababa, Ethiopia, 2014. PLWH, person living with HIV; HIV, human immunodeficiency virus; ART, antiretroviral therapy.

A follow-up study was carried out at selected public health facilities (3 hospitals and 2 health centers) in Addis Ababa that were selected by lottery between June 2012 and October 2013. A total of 360 HIV-positive women, including 190 newly initiated women and 170 ART-naïve women, were included in the study (Figure 1).

The sample size was computed using a 2-proportion formula with the assumption that the proportion of fertility intentions among HIV-positive women who were ART-naïve and 12 months after ART initiation would be 26.6 and 21.5%, respectively [30], with a minimum detectable odds ratio (OR) of 2 at the 5% level of significance, power of 80%, and a 20% non-response rate; based on this, a total sample size of 407 was calculated.

### Sampling procedures

Women were eligible if at enrollment they were between 18 and 49 years of age, had been sexually active in the past 3 months, had not undergone tubal ligation or hysterectomy, and had not diagnosed as permanently infertile. Further inclusion criteria included not being pregnant, being willing for their records to be reviewed; and either being newly initiated on ART or ART-naïve.

A total of 5,678 patients (men and women) were receiving care at the selected sites, of whom 3,118 were women of reproductive age. Of these women, 407 were eligible and 360 (including 190 ART-initiated and 170 ART-naïve) participated (Figure 1).

The study was linked with the routine activities of each health institution. The recruitment procedure was conducted using the appointment log book, and screening was conducted every day. Women who came for follow-up and for ART initiation were identified separately and screened for their eligibility according to parameters such as age, ART initiation, being ART-naïve, World Health Organization (WHO) staging, and other factors affecting eligibility for inclusion. An identification number was given to the eligible women for use as a sampling frame for the selection of study participants using systematic random sampling.

Patients were enrolled only after they provided informed consent. No financial remuneration was offered to participants. Once enrolled, a baseline questionnaire was administered in a private room, while the participants waited to see ART clinic staff.

#### Follow-up procedure

The follow-up for both groups (women on ART and ART-na- ïve women) was scheduled in accordance with the respective institutional schedule of 2 follow-up visits over a period of 1 year. The ART-initiated women were interviewed during refill (treatment collection), and the ART-naïve women were interviewed during their appointment for CD4 cell testing. The same tool was used both times and collected the same information as the baseline survey, except for socio-demographic factors and the WHO stage of disease, which cannot change over time. For ease of follow-up, a unique identification mark labeled with the date of the baseline survey and the scheduled time of the next appointment was affixed to the patient’s history book and the questionnaire was collected as baseline data. A study staff member reminded them of their next interview time when they collected their drugs or came for other follow-up.

#### Tracking procedure

During the baseline interview, the contact details of each participant (cell phone, other contact address such as a relative’s or friend’s cell phone, the unique ART number, and next scheduled appointment) were recorded. In addition, detailed directions to the patient’s home, place of work, and other convenient places were recorded. Every effort was made to minimize loss to follow-up. For example, if a woman missed her appointment, phone contact was made, a home visit for an interview was made for those who were willing to be visited at home, or another convenient appointment time or place was decided upon.

When a client could not be traced for 3 months either by 4 calls and/or 1 home visit, we failed to locate the home, or we found that she relocated to an inaccessible place, she was considered to be lost to follow-up.

### Data collection procedure

Data were collected using interviewer-administered structured questionnaires. The questionnaire was translated into the local language (Amharic) by experts in both languages and back-translated to English by another person to ensure consistency and accuracy. Data collectors were all woman nurses recruited based on their previous experience, such as previous training on ART and HIV counseling and fluency in the local language. Training was given for 5 consecutive days on interview techniques, sampling and ethical issues, the importance of the safety of participants and interviewers, minimization of under-reporting and maintaining confidentiality. A pre-test of the questionnaire was conducted in selected ART units that were not included in the main study. The data collection process was closely supervised by the principal investigator and a supervisor.

The baseline questionnaire included information on socio-demographic characteristics (age, gender, marital status, monthly income, residence, religion), HIV testing history and disclosure of HIV status, fertility intentions, current use of contraception, and number of living children. The follow-up questionnaires included similar questions to that of the baseline.

### Measurement and operational definition of variables

#### To assess changes in the fertility intentions of human immunodeficiency virus-positive women

The outcome of interest was evaluated in 2 visits through the following question: ‘Do you want to have a child in the future?’ Women were free to answer ‘yes,’ ‘no,’ or ‘do not know.’ Fertility intention was determined based on the woman’s reports at baseline and at the 12-month follow-up visit.

A change in fertility intentions occurred if a woman reported wanting no more children at baseline and reported wanting to have more children at the 12-month follow-up visit. A change in fertility intentions occurred if a woman reported wanting more children at baseline and then reported wanting no more children at follow-up; this group included those who reported that they were undecided or had adopted a birth control method at the follow-up visit. No change in fertility intentions took place if a woman reported wanting more children at the baseline visit, along with no contraceptive method use, and reported wanting more children or gave the same response at the 12-month visit. No change in fertility intentions took place when women reported no fertility intention and the use of birth control at the baseline, and gave the same responses at the 12-month follow-up visit.

A concordant response at the baseline visit and at the 12-month follow-up visit was coded as no change “0”, while a discordant response indicated a change in fertility intentions and was coded as “1”.

### Analysis

The pre-coded responses were double-entered into Epi Info^TM^ version 3.5.2 (Centers for Disease Control and Prevention, Atlanta, GA, USA) and exported to SPSS version 20 (IBM Corp., Armonk, NY, USA) for data checking, cleaning, and analysis.

We compared the baseline characteristics of ART-initiated and ART-naïve women using the McNamara test for categorical variables. The McNamara test showed a significant difference between the baseline and 12-month measurements of fertility intentions (p<0.001). Based on these findings, all concordant (similar) responses were combined, as were the discordant (different) responses, to form the outcome variable that was ultimately assessed in this study.

Findings regarding fertility intentions are presented using descriptive statistics. The strength of the associations between changes in fertility intentions and socio-demographic, reproductive health, and clinical factors were assessed using ORs with 95% confidence levels (CIs). Logistic regression analysis was done to assess the relative effect of independent variables and fertility intentions, and the adjusted results (adjusted OR [aOR]) are presented.

### Ethical considerations

The research was approved in terms of its scientific and ethical integrity by the institutional review board of the College of Health Sciences, Addis Ababa University. Written permission was obtained from the health bureau of the Addis Ababa city government. Consent was obtained from the respective unit heads at each health institution. Verbal consent was also obtained from individual clients. Sufficient information was given to each participant for them to make an informed decision. Confidentiality was strictly maintained for each piece of information and all interviews were conducted in a strictly private place. At the end of the interview, general information, referrals, and follow-up linkage were provided for those in need.

## RESULTS

At the baseline, a total of 407 clients were interviewed. After 12 months, 360 of the 407 clients were ultimately included in the study. Forty-seven clients were not included in the study due to the following reasons: they were lost to follow-up (6), died before the study ended (2), were ART-naïve clients who started ART after the study commenced (7), did not volunteer to participate in the study (10), were not from the study site (3), only made 1 visit during the monitoring period (3), became critically ill during data collection (3), transferred out (5), or were older than 49 years (8).

### Socio-demographic characteristics of the study subjects

At the baseline visit, 108 (30.0%) and 100 (27.8%) subjects were within the age ranges of 30-34 and 25-29 years, respectively. The mean with standard deviation (SD) age of the subjects was (31.0±5.6). ART-naïve women had more living children, with a mean (SD) of 1.4 (1.3) than ART-initiated women, who had a mean (SD) of 1.2 (1.2) numbers of living children.

The predominant ethnic group and religion were Amhara and Orthodox Christians 57.5 and 84.2%, respectively. Of the participants, 141 (39.2%) had completed grades 1-8; 202 (56.1%) were economically dependent or received a monthly income of less than 500 Ethiopian birr (26 US dollars), and 104 (28.9%) were unemployed. About half of the participants (51.1%) were married or cohabited, and 23.3% were single (Table 1).

### Reproductive, clinical, and fertility intentions of human immunodeficiency virus-positive women (baseline vs. at 12-month follow-up)

Of the participants, 40.8% reported fertility intentions at the baseline visit, while 48.3% reported fertility intentions at the 12-month follow-up. At the baseline visit, 42.1% of the ART-initiated women and 39.4% of the ART-naïve women reported desiring more children. At the 12-month follow-up, this proportion increased to 55.8% (106 of 190) among the ART-initiated women and 40.0% (68 of 170) among the ART-naïve women (Table 2).

Of the 147 women who reported desiring children at the baseline visit, 31.1% said that they intended to have a child (to give birth) within 12 months, while the majority 35.1% had not yet decided when to have children, but instead reported that the timing would depend on ‘God’s will’ or that they ‘did not know’. In contrast, at the 12-month follow-up, 38.5% and 20.7% of the women who reported desiring children stated that wanted to have children within 12 months and between 12 and 24 months, respectively. Sixty (35.1%) did not say anything regarding when they intended to have children. In terms of number of children, 82.3% (121 of 147) and 83.3% (145 of 174) reported the desire to have 2 or more children in the future at the baseline visit and at the follow-up visit, respectively.

The prevalence of contraceptive use decreased from 53.9% at the baseline to (48.3%) at the 12-month follow-up. Only 6.7% and 7.5% reported dual contraceptive use at baseline and at the 12-month follow-up, respectively (Table 2).

### Predictors of change in fertility intentions

The adjusted analysis indicated that changing from wanting no more children at baseline to wanting another child at the 12-month follow-up was significantly associated with ART initiation (aOR, 2.47; 95% CI, 1.20 to 5.20) and marital status, with single (aOR, 5.33; 95% CI, 1.10 to 25.92) and married (aOR, 6.35; 95% CI, 1.44 to 27.99) being more likely to report fertility intentions than divorced/widowed women. Additionally, the presence of living children (1 child: aOR, 0.33; 95% CI, 0.13 to 0.85; 2 or more children: aOR, 0.36; 95% CI, 0.15 to 0.91) led women to be less likely to report changes in their fertility intentions than women without children. Family influence (aOR, 3.67; 95% CI, 2.16 to 6.22), disclosure of HIV status to the husband (aOR, 1.96; 95% CI, 1.10 to 3.60), and being a housewife (aOR, 3.60; 95% CI, 1.16 to 10.91) were also predictors of change in fertility intentions. In contrast, educational status, monthly income, the partner’s desire to have children (aOR, 2.10, 95% CI, 0.83 to 5.01), and the partner’s HIV test result (aOR, 0.64; 95% CI, 0.22 to 1.86) had no association with changes in the fertility intentions of women living with HIV (Table 3).

### Reasons for the fertility intentions of human immunodeficiency virus-positive women

Of the 360 participants, 40.8% (n= 147) vs. 48.3% (n= 174) reported fertility intentions at the baseline visit and at the 12-month follow-up, respectively. The reasons for positive fertility intentions were related to medical concerns and were socially and culturally oriented, as shown in Table 4. The most common reasons given for wanting a child at baseline and at the 12-month follow-up, respectively, were related to ART use 70.7% (104 of 147) vs. 54.0% (94 of 174), followed by improvements in the participant’s health condition 6.8% vs. 27.6%, and children being an important part of marriage 7.5% vs. 6.3%. A small percentage 4.8% at baseline and 2.9% at the 12-month follow-up thought that it was just due to recent marriage, stated that they wanted a child due to the husband’s influence 4.1% vs. 2.9%, and some were concerned with replacing themselves.

## DISCUSSION

In this study, we assessed changes in fertility intentions between the time of ART initiation and a 12-month follow-up, as well as factors associated with fertility intentions. Our findings from this 2-round analysis indicated a change in the proportion of HIV-positive women with positive fertility intentions from 40.8% at the baseline visit to 48.3% at the 12-month follow-up. This finding is similar to what other studies have found in sub-Saharan Africa [8,30,31]. However, they contrast with the findings of a recent cross-sectional study in South Africa [20], which may have resulted from differences in the study design.

There is no argument that childbearing is as important to persons living with HIV as it is to their non-infected counterparts [32]. African social and cultural values dictate that having children is key to the identity and social status of both women and men, including persons living with HIV. In agreement with this, our findings documented that the majority of women reported that they considered children to be an important part of marriage. It was also reported that childbearing helps to secure a marriage and the continuity of one’s lineage, as stated by many families. These findings are also substantiated by a previous study that found that women on ART quickly became pregnant even before their CD4 counts improved both because they wanted to fulfill their social role as women and because they wanted to prove that they were healthy [23].

ART use, together with other factors, was found to be a significant predictor of fertility intentions among HIV-positive women in the study area of Addis Ababa, where the total fertility rate is 2.4 children per reproductive-age woman [4]. ART users were more likely than ART-naïve women to change their fertility intentions from wanting no more children at the baseline visit to wanting another child at the follow-up visit that took place 12 months after ART initiation. The effect was statistically significant after controlling for potential confounders of fertility, such as the number of living children, age, income, and socio-demographic conditions. Furthermore, it was identified that changes in fertility intentions were associated with the number of living children women had and marital status. This is similar to previous reports from subSaharan Africa [31].

In comparison to the baseline, the contraceptive use of women decreased at the 12-month follow-up visit, although at both time points, most study subjects reported hormonal contraceptive use, which is effective in preventing unintended pregnancy. However, a low proportion of dual-method use (6.7%, n= 13 vs. 7.5%, n= 14) was reported at the baseline and at the 12-month follow-up, respectively. This finding is similar to that reported (15.0%) in a previous study from sub-Saharan Africa [22]. This low rate was found even though the use of 2 contraceptive methods (a condom with another method) is safe for preventing unintended pregnancy and HIV [33,34]. Our findings reinforce the results from other studies, and the low prevalence of dual contraceptive use indicates the low acceptance of condom use in sub-Saharan Africa due to sociocultural factors and insufficient information [35]. The study population might be at risk of acquiring sexually transmitted infections, including drug-resistant strains of HIV.

Changing from wanting no more children at the baseline visit to wanting another child was found to be likely to occur soon after ART initiation among HIV-positive women. Thus, family planning should be an immediate priority for providers and should be discussed regardless of ART initiation, in order to allow women living with HIV in the area to work toward their fertility and contraceptive goals in a safe manner.

A strength of this study is that it tracked a cohort of both ART users and ART-naïve women for over a year. A potential limitation of this study is the relatively short duration of follow-up, the fact that we did not include HIV-negative women, and the limitation of the study to public health facilities. Our design is also potentially subject to social desirability bias, as the study outcome was self-reported fertility intentions. To minimize these factors, interviewers were not from the same institutions, training was given, and data collection was strictly supervised.

In conclusion, ART use was found to be a significant predictor of change in fertility intentions, suggesting that additional effort is necessary to integrate family planning and HIV services to safely address the fertility goals of HIV-positive women in the study area. Furthermore, a considerable proportion of women still did not consistently use a dual-contraceptive method, which remain a cause for concern.

Finally, future research should investigate the potential influence of ART on the incidence of pregnancy and changes in preventive practices with the involvement of partners.

## Figures and Tables

**Table 1.** Socio-demographic characteristics of HIV-positive women in Addis Ababa, Ethiopia

Characteristics	ART users (n=190)	ART-naïve (n=170)	Total
Age (yr)			
18-24	22 (11.6)	16 (9.4)	38 (10.6)
25-29	51 (26.8)	49 (28.8)	100 (27.8)
30-34	59 (31.1)	49 (28.8)	108 (30.0)
35-39	47 (24.7)	46 (27.1)	93 (25.8)
40-49	11 (5.8)	10 (5.9)	21 (5.8)
Mean士SD	31.0±5.6	31.2±5.5	31.1±5.5
Ethnicity			
Amhara	112 (58.9)	95 (55.9)	207 (57.5)
Oromo	39 (20.5)	52 (30.6)	91 (25.3)
Guragea	21 (11.1)	13 (7.6)	34 (9.4)
Tigrea	11 (5.8)	8 (4.7)	19 (5.3)
Others (Selte, Hadya, Sidama)	7 (3.7)	2 (1.2)	9 (2.5)
Religion			
Orthodox Christian	159 (83.7)	144 (84.7)	303 (84.2)
Muslim	19 (10.0)	14 (8.2)	33 (9.2)
Protestant	12 (6.3)	12 (7.1)	24 (6.7)
Education			
Illiterate/informal education	39 (20.5)	39 (22.9)	78 (21.7)
Grades 1-8	70 (36.8)	71 (41.8)	141 (39.2)
Grades 9-12	70 (36.8)	47 (27.6)	117 (32.5)
Grades 12 and above	11 (5.8)	13 (7.6)	24 (6.7)
Monthly income in birr^1^			
No income/dependent/<500	105 (55.3)	97 (57.0)	202 (56.1)
501-3,000	78 (41.0)	69 (40.6)	147 (40.8)
>3,000	7 (3.7)	4 (2.4)	11 (3.1)
Occupation			
Unemployed	58 (30.5)	46 (27.1)	104 (28.9)
Housewife	31 (16.3)	24 (14.1)	55 (15.3)
Daily laborer	21 (11.1)	22 (12.9)	43 (11.9)
Merchant	18 (9.5)	8 (4.7)	26 (7.2)
Government employee	12 (6.3)	19 (11.2)	31 (8.6)
Private businesses	50 (26.3)	51 (30.0)	101 (28.1)
Marital status			
Single	49 (25.8)	35 (20.6)	84 (23.3)
Married/cohabiting	92 (48.4)	92 (54.3)	184 (51.1)
Divorced/widowed	49 (25.8)	43 (25.3)	92 (25.6)

Values are presented as number (%).

HIV, human immunodeficiency virus; ART, antiretroviral therapy; SD, standard deviation.

1 One US dollar = 19.20 Ethiopian birr.

**Table 2.** Reproductive, clinical, and fertility intentions of HIV-positive women (baseline vs. 12-month follow-up) in Addis Ababa, Ethiopia

Description	Baseline			At 12 mo follow-up		
	ART-initiated (n=190)	ART-naïve (n=170)	Total	ART users (n=190)	ART-naïve (n=170)	Total
No. of living children						
0	66 (34.7)	48 (28.2)	114 (31.7)	66 (34.7)	48 (28.2)	114 (31.7)
1	50 (26.3)	52 (30.6)	102 (28.3)	50 (26.3)	52 (30.6)	102 (28.3)
2+	74 (38.9)	70 (41.2)	144 (40.0)	74 (38.9)	70 (41.2)	144 (40.0)
Desire to have more children						
Yes	80 (42.1)	67 (39.4)	147 (40.8)	106 (55.8)	68 (40.0)	174 (48.3)
No	110 (57.9)	103 (60.6)	213 (59.2)	84 (44.2)	102 (60.0)	186 (51.7)
No. of children desired						
1	13 (16.2)	13 (19.4)	26 (17.7)	16 (15.1)	13 (19.1)	29 (16.7)
2	50 (62.5)	45 (67.2)	95 (64.6)	68 (64.2)	46 (67.6)	114 (65.5)
3+	17 (21.2)	9 (13.4)	26 (17.7)	22 (20.8)	9 (13.2)	31 (17.8)
Time to have more children (mo)						
>12	24 (29.6)	22 (32.8)	46 (31.1)	41 (38.7)	26 (38.2)	67 (38.5)
12-24	14 (17.3)	26 (38.8)	39 (27.0)	13 (12.3)	23 (33.8)	36 (20.7)
24-36	5 (6.2)	5 (7.5)	10 (6.8)	6 (5.7)	4 (5.9)	11 (6.3)
Undecided (‘do not know' and ‘God's will')	38 (46.9)	14 (20.9)	52 (35.1)	46 (43.4)	15 (22.1)	60 (34.5)
Contraceptive use						
Yes	87 (45.8)	107 (62.9)	194 (53.9)	70 (36.8)	104 (61.2)	174 (48.3)
No	103 (54.2)	63 (37.1)	166 (46.1)	120 (63.2)	66 (38.8)	186 (51.7)
Type of contraceptives						
Hormonal	43 (49.4)	61 (57.8)	104 (54.1)	33 (47.1)	56 (54.3)	90 (51.4)
Condom	40 (46.5)	37 (34.4)	77 (39.3)	32 (45.7)	39 (37.1)	71 (40.6)
Dual protection	4 (44.6)	9 (8.3)	13 (6.7)	5 (7.1)	9 (8.6)	13 (7.5)
Who influenced you (n=107)						
My family	37 (68.5)	33 (62.3)	70 (65.4)	34 (64.2)	31 (60.8)	65 (62.5)
His family	17 (31.5)	20 (37.7)	37 (34.6)	19 (35.8)	20 (38.5)	39 (37.5)
Partner tested (n=184)						
Yes	62 (67.4)	82 (89.1)	144 (78.3)	67 (72.8)	82 (89.1)	149 (81.0)
No	30 (32.6)	10 (10.9)	40 (21.7)	25 (27.2)	10 (10.9)	35 (19.0)
Partner's test result (n=149)						
Positive	45 (72.6)	60 (73.2)	105 (72.9)	51 (76.1)	60 (74.5)	111 (74.5)
Negative	17 (27.4)	22 (26.8)	39 (27.1)	16 (23.9)	22 (26.8)	38 (25.5)
Disclosed HIV status (n=184)						
Yes	49 (53.3)	57 (53.8)	106 (57.6)	57 (62.0)	62 (67.4)	119 (64.7)
No	43 (46.7)	35 (38.0)	78 (42.4)	35 (38.0)	30 (32.6)	65 (35.3)

Values are presented as number (%).

HIV, human immunodeficiency virus; ART, antiretroviral therapy.

**Table 3.** Adjusted logistic regression analysis of changes in fertility intentions among HIV-positive women at the 12-month follow-up visit, Addis Ababa, Ethiopia

Variables	Change in fertility intentions		aOR (95% CI)
	Yes (n=41)	No (n=319)	
HAART use			
ART-naïve	11 (26.8)	159 (49.8)	1.00 (reference)
ART-initiated	30 (73.2)	160 (50.2)	2.47 (1.20, 5.20)^***^
Marital status			
Widowed/divorced	3 (7.3)	89 (27.9)	1.00 (reference)
Single	13 (31.7)	71 (22.3)	5.33 (1.10, 25.92)^*^
Married	25 (61.0)	159 (49.8)	6.35 (1.44, 27.99)^*^
Occupation			
Dependent	6 (14.6)	98 (30.7)	1.00 (reference)
Housewife	13 (31.7)	42 (13.2)	3.60 (1.16,10.91)^*^
Daily laborer	3 (7.3)	40 (12.5)	1.10 (0.24, 4.80)
Merchant	6 (14.6)	20 (6.3)	2.40 (0.52,10.81)
Government employee	3 (7.3)	28 (8.8)	1.30 (0.26, 6.61)
Private business	10 (24.4)	91 (28.5)	1.22 (0.36, 4.20)
Monthly income in birr^1^			
No income/dependent/<500	18 (43.9)	184 (57.7)	1.00 (reference)
501-3,000	19 (46.3)	128 (40.1)	1.41 (0.57, 3.46)
>3,000	4 (9.8)	7 (2.2)	3.49 (0.70,17.70)
Education			
Illiterate/informal education	9 (21.7)	69 (21.6)	1.00 (reference)
Grades 1-8	18 (43.9)	123 (38.6)	0.96 (0.40, 2.44)
Grades 9-12	11 (26.8)	106 (33.2)	0.78 (0.27, 2.26)
Grades 12 and above	3 (7.3)	21 (6.6)	1.10 (0.22, 5.10)
No. of living children			
0	22 (53.7)	92 (28.8)	1.00 (reference)
1	9 (22.0)	93 (29.2)	0.33 (0.13, 0.85)^*^
2+	10 (22.4)	134 (42.0)	0.36 (0.15, 0.91)^*^
Family influence (n=360)			
No	24 (58.5)	232 (72.7)	1.00 (reference)
Yes	17 (41.5)	87 (24.3)	3.67 (2.16, 6.22)^***^
Disclosed HIV status to partner (n=184)			
No	3 (12.0)	62 (39.0)	1.00 (reference)
Yes	22 (88.0)	97 (61.0)	1.96 (1.10, 3.60)^*^
Partner wants to have children (n=184)			
No	8 (32.0)	78 (49.1)	1.00 (reference)
Yes	17 (68.0)	81 (50.9)	2.10 (0.83, 5.01)
Partner tested (n=184)			
No	7 (28.0)	28 (17.6)	1.00 (reference)
Yes	18 (72.0)	131 (82.4)	0.55 (0.21,1.44)
Test result of partner (n=149)			
Negative	6 (33.2)	32 (24.4)	1.00 (reference)
Positive	12 (66.7)	99 (75.6)	0.64 (0.22,1.86)

Values are presented as number (%).

HIV, human immunodeficiency virus; HAART, highly active antiretroviral therapy; ART, antiretroviral therapy; aOR, adjusted odds ratio; CI, confidence interval.

1 One US dollar = 19.20 Ethiopian birr.

* p<0.05,

*** p<0.001.

**Table 4.** Reasons for wanting to have children in the future (before and after)

Reasons for wanting to have children	Baseline (n=147)	At 12 mo follow-up (n=174)	
Taking ART	104 (70.7)	94 (54.0)
Health condition improved	10 (6.8)	48 (27.6)
Recent marriage	7 (4.8)	5 (2.9)
Husband insisted on it	6 (4.1)	5 (2.9)
Health workers' advice	2 (1.4)	4 (2.3)
To replace myself / original desire for childbearing unchanged by HIV	5 (3.4)	5 (2.9)
Current child needs siblings/do not want to leave my child alone	2 (1.4)	2 (1.1)
I like children (children are an important part of marriage, either for present or future marriage)	11 (7.5)	11 (6.3)
		

Values are presented as number (%).

ART, antiretroviral therapy; HIV, human immunodeficiency virus.
